# La Grippe-Its Manifestations, Complications and Treatment

**Published:** 1899-10

**Authors:** W. W. Grube

**Affiliations:** Toledo, Ohio, Professor of physiology and clinical medicine, Toledo Medical College, Toledo, Ohio


### Abstracts.

---

### LA GRIPPE—ITS MANIFESTATIONS, COMPLICATIONS AND TREATMENT.

By W. W. GRUBE, A. M., M. D., of Toledo, Ohio,

Professor of physiology and clinical medicine, Toledo Medical College, Toledo, Ohio.

[Abstract from the *Journal of the American Medical Association*, March 25, 1899.]

PROFESSOR GRUBE sees no reason why the intelligent observer need err in his diagnosis of la grippe; he believes that the intensity of the catarrhal symptoms the great prostration, and tardy convalescence form a typic clinical picture. Though the catarrhal symptoms are usually limited to the respiratory mucous membrane, they are not always so, and in the writer's experience the invasion of the mucous membrane of the digestive tract has been quite frequent. Not alone mucous membrane, but a part of all of the cerebro-spinal axis has been invaded.

In many cases the so-called complications are simply an extension and aggravation of the catarrhal or inflammatory condition; thus an extension of the usual inflammatory condition of the throat through the Eustachian tube produces middle-ear complications; the bronchitis, too, may extend and become capillary, or even a pneumonitis may result. So we believe that in the so-called abdominal form with severe gastro-enteric catarrh, it may extend by *contiguity* and inaugurate a general peritonitis. Upon this theory alone can we explain the supervention of a severe general peritonitis in a case under our care, now happily terminating in convalescence.

The patient was a girl of 11 years who had never been seriously ill before. Twenty-four hours after the illness began, she had, besides the usual alarming symptoms of la grippe, a high temperature, wild delirium, constant emesis, frequent and copious discharge of feces and urine. The appropriate remedies were prescribed, the vomiting ceased and she rested; but on the third or fourth day she developed symptoms of peritonitis, abdominal pain, hardness and some tympanites. Calomel was prescribed, twenty grains divided into four powders, one every three hours; also the usual turpentine stupes and morphia to quiet pain. The next day, finding no improvement, but rather aggravated symptoms, green vomit, bowels not moved—a very gloomy prognosis was given, and at the family's request a consulting physician was called, who concurred in diagnosis and prognosis, and had nothing more to suggest. On the writer's return in the evening, however, he decided in view of the great mortality of these cases by the routine treatment, to try the local application of a mustard poultice; also, for their germicidal, antiseptic, and healing qualities, he gave internally hydrozone diluted, in frequent doses, alternating with doses of glycozone. In twenty-four hours there was slight improvement. In forty-eight hours the patient was decidedly better. Improvement continued and the girl was so well February 21st, that she was dismissed as cured.

Perhaps the most common complication in children is the middle-ear inflammation caused by extension of the pharyngeal catarrh up the Eustachian tube into the tympanum. In the case of a child six months old, recently under our care, we had a middle-ear complication, in which the pain was controlled by the usual methods and by the instillation into the aural canal of a few drops of cocaine solution. After suppuration occurred, however, the canal was cleansed by hydrozone solution (warm) and a piece of absorbent cotton saturated with glycozone used as a dressing by inserting it into the canal. As the ear complications sometimes prove very serious, it is gratifying to know that in the above remedies we have a safe, speedy and effectual method of cure. We believe also that, if these cases were seen early, by proper treatment the extension and consequent complications might be prevented. In a little girl with severe tonsillitis and pharyngitis we are now spraying the throat with diluted hydrozone and applying glycozone with such marked benefit that on this, the third day of treatment, she is almost well.

In concluding Professor Grube states:—"I cannot refrain from referring to the case of a prominent city official who had an unusually severe attack of la grippe. All the structures of the nasal cavities were involved in a severe acute catarrh, which progressed at the stage of suppuration. Enormous quantities of pus were secreted, and the location and intensity of the pain led us to fear involvement of the antrum. However, the free use of hydrozone solution by spraying and the application of glycozonesoon cleared up the cavity, and in a few days complete cure resulted."

---

### A SUBSTITUTE FOR SUGAR IN DIABETES.

NOTWITHSTANDING the large number of remedies brought forward from time to time for the cure of diabetes, the dietetic treatment still continues to occupy the most important part in the management of this disease. In view of the fact that in severe cases the use of starches and sugars is absolutely interdicted, much ingenuity has been exercised in devising substances which would replace these foods. While the attempts to produce substitutes for starchy foods have not been very successful, much has been accomplished in the discovery of artificial sweetening agents, which enable the patient to gratify his craving for sugar, without producing the harmful effects of the latter. Among the sugar substitutes sycose represents the latest stage in the evolution of a perfect product of this kind. It has a sweetening power of 550 times greater than that of cane sugar. Owing to its chemical purity, its freedom from the inert matter found in other substitutes for sugar, its pure taste and solubility, it is eminently adapted for medicinal use. Sycose is therefore well worthy of a careful trial in the treatment of diabetes and of all other diseases in which the use of ordinary sugar in any form is contraindicated.

---

### CONGENITAL NASAL ATRESIA.

DR. HAL FOSTER (*The Laryngoscope*, May, 1899,) reports an interesting case of congenital nasal atresia in a boy eight years old. According to the mother's statement the nose was closed at the child's birth. There was no other deformity. On examination a definite white fibrous membrane stretching between the septum and the side wall of the nostril was found. Occlusion existed on one side only. As there was a history of syphilis in the boy's father specific medication was resorted to for eight weeks. At the end of that time the membrane was divided by a surgical drill, and the galvano-cautery applied, after which a small Asch nasal splint was inserted and held in place by a piece of plaster. Antiseptic sprays were used three times daily, and europhen blown into the nose every morning. Under this treatment a perfect cure was effected.

